# To Improve the Solubility of India Rubber

**Published:** 1864-02

**Authors:** 


To Improve the Solubility of India Rubber.—Caoutchouc (virgin gum) becomes more soluble in oil of turpentine or benzine, if it has previously been macerated for some time with ammonia, until perfectly decolorized. When it is then washed with water, and dried, it will dissolve more readily in the menstrua named.—*Am. Drug. Circ.*

